
JOURNAL INFORMATION
==============================
NLM Title Abbreviation: Insights Imaging
Journal ID: Insights Imaging
NLM Journal ID: 101532453

Insights into Imaging

EISSN: 1869-4101
Publisher: Springer

ARTICLE INFORMATION
==============================
PMCID: PMC7201111
PMID: 32377971
DOI: 10.1186/s13244-020-00851-0
Article ID: 851
Article version: 2
Subjects: Meeting Abstracts

ECR 2020 Book of Abstracts

Vienna, Austria. 15 March 2020

Electronic publication date: 2020 May 5
Publication date: 2020 May
Volume: 11
Issue: Suppl 1
Issue sponsor: This publication was not supported by sponsorship.
Electronic Location ID: 34
Copyright: © The Author(s) 2020
License: Open AccessThis article is distributed under the terms of the Creative Commons Attribution 4.0 International License (http://creativecommons.org/licenses/by/4.0/), which permits unrestricted use, distribution, and reproduction in any medium, provided you give appropriate credit to the original author(s) and the source, provide a link to the Creative Commons license, and indicate if changes were made.
License URL: https://creativecommons.org/licenses/by/4.0/

Conference name: European Congress of Radiology 2020
Conference Acronym: ECR 2020
Conference location: Vienna, Austria
Conference date: March 11 - March 15 2020
issue-copyright-statement: © The Author(s) 2020

==============================
Publisher’s Note

Springer Nature remains neutral with regard to jurisdictional claims in published maps and institutional affiliations.

